# Symptom-Based Contextual Models of Cognition and Judgment for Resolving Biased Decision Making Under Uncertainty

**DOI:** 10.3390/e28060623

**Published:** 2026-06-01

**Authors:** Gueorgui Petkov

**Affiliations:** 1 Atomic Physics Department, Faculty of Physics, Sofia University “St. Kliment Ohridski”, 5 James Bourchier Blvd., 1164 Sofia, Bulgaria; gipetkov@uni-sofia.bg or gipetkov@npp.bg; Tel.: +359-886518796; 2 Kozloduy Nuclear Power Plant, 3321 Kozloduy, Bulgaria

**Keywords:** socio-technical system, symptoms, bias, context, cognition, judgment, uncertainty, risk, ambiguity aversion

## Abstract

This article presents a contextual entropic model for understanding, explaining, and resolving cognitive biases associated with thinking under uncertainty. This article applies a unique performance evaluation of teamwork method, enabling a qualitative and quantitative assessment of symptom-based context and ensuring a clear and rational interpretation of decision making in ambiguous and risky situations. This human reliability assessment method also contributes to a deeper understanding of the iterative and complementary nature of cognition and decision making. The main idea is the quantum-like understanding of the dual image of a symptom as a wave and a bit of information in thought processes. Thus, each context alternative is identified by a unique combination of three-valued states of the socio-technical system at discrete points in time—recognized, unrecognized, and unrecognizable. Judgment is a wave-like process driven by the interfering sum of the amplitudes of symptom recognition shifts. By modeling stepwise cognitive processes by adding and subtracting symptoms or stimuli, we can estimate and compare the likelihood ratios between biased judgments. This article presents three canonical filters used to improve cognitive theories and decision-making methods—Ellsberg’s two-color, three-color, and four-color paradoxes—to demonstrate the power of the symptom-based context model.

## 1. Introduction

A person can be irrational and erroneous in thoughts, choices, and actions. The most trivial and plausible reasons for these errors are the lack of knowledge, abilities, experience, or chance, which are individual for each person in a specific cognizable and incognizable situation. To reduce the likelihood of error and avoid biased, irrational and risky judgments, we must know or at least be able to imagine and model the construction, implementation mechanism and control of thought processes.

The most prevalent version of the rational choice is the theory of expected utility (EU) [1]. Despite its popularity, this theory is not based on in-depth causal, “conclusive, explanatory models or theories” of the processes of cognition and decision making. But rather, its proponents and followers are looking for interesting effects leading to “a rich set of phenomena” [2] through paradoxical thinking or fitting approximate formulas using increasingly complex mathematical constructs. In this sense, the only way to judge the success of a choice hypothesis is through empirical testing and validating. However, they can easily be challenged by the experimental results of behavioral economics and psychology. It is therefore advisable and necessary to use the experience of physics, which tries to synthesize as many phenomena as possible in one or in as few theories as possible [3].

Cognitive process is wavy, diffuse, vague, and ambiguous, and therefore it must be modeled as dispersed and non-dispersed, stochastic and non-stochastic, because human actions occur consistently and causally, but also accidentally. How do you resolve these contradictions to understand and evaluate judgments? The most prevalent version of treating a judgment or “rational choice without self-relevant consequences” is the sure-thing (ST) principle in the framework of the EU theory. But there are some problems, such as Ellsberg’s and Machina’s paradoxes, conjunction, and disjunction fallacies, etc., which introduce dissonance and contradictory interpretations in a judgment under uncertainty, preventing a clear picture of the cognitive process from being obtained. This happens despite the use of advanced mathematical models and the borrowing of some universal ideas from physics, such as quantum mechanics, to explain nature to balance true, pseudo and false randomness.

A scientific approach to solving the problem of human error also requires describing and modeling the processes of cognition, decision making, and action by measuring, evaluating, and analyzing everything that can be theoretically inferred, determined, or attributed to empirical frequencies and situations that are unrecognizable, indefinite, ambiguous, and risky. All these objective and subjective factors and conditions are related to the context of thought and manual processes, which, we hope, can be described qualitatively and quantitatively with the help of probability.

Knight [4] tried to address this issue by distinguishing between measurable uncertainty or risk, presented as objectively known probabilities (OP), and immeasurable uncertainty or ambiguity, which was determined in the extended subjective expected utility (SEU) theory [5] by subjectively personal judged probabilities (SP). Introducing vague personal probabilities, at the same time, Savage was trying to eliminate vagueness from the rational choice theory. This fuzziness in the theory of rational choice and the deviations between OP and SP still seem inexplicable, although the relativity of cognition and judgment has long been known, and as Plato argues: “We do not see things as they really are, but merely as we see them.” But the goal of cognitive research is namely to bridge the gap between observed phenomena, objectively existing and subjectively recognizing, and their corresponding unexplained results [6].

A lot of models have been proposed to generalize SEU and to accommodate ambiguity preferences in Ellsberg’s [7] and Machina’s paradoxes [8,9] including the two-stage model in [10], Choquet expected utility in [11], maximin expected utility in [12], Bayesian approach-based model in [13], cumulative prospect theory in [14], α-maximin expected utility model in [15], the smooth model of ambiguity aversion in [16], variational preferences model in [17], vector expected utility in [18], expected uncertainty utility in [19], and two-stage evaluation in [20], among others. However, all these models fail and struggle to cope with all the above paradoxes, as well as other examples and filters of biased thinking and judgment, highlighting the need for improved understanding.

It has been proven that biased judgments arise from uncertainty, and their root causes are the lack of knowledge and the unattainable balance between determinism and randomness in our understanding of nature and humans. This uncertainty is resolved by dividing, decomposing, and simplifying the socio-technical system (STS) into subsystems and reducing the dependent factors to a set of independent ones. However, the impact of these factors on STS is studied separately and outside the context of the entire system, and if the context is not considered, uncertainties can be amplified and resonate many times over. The context must consider all states and alternatives for the development of the STS and therefore can be represented as a set of objectively arising stimuli (symptoms) and their subjective images for a person in a specific situation. To reduce uncertainty, it is necessary to minimize risk and reduce ambiguity. Therefore, to explain and overcome the aversion to uncertainty and prevent biased and erroneous judgments, an entropy model of the context of cognition in STS is needed that tracks the deterministic–probabilistic interaction of symptoms in the process of their recognition.

A practical approach to dealing with misjudgments must be based on scientific knowledge, reliable statistics, and robust heuristics, effectively utilizing the interaction of deterministic and probabilistic models. It also requires describing and modeling the context of thought processes, linked to the objective and subjective images of the STS’s symptoms in each situation [21]. This can be achieved by measuring and assessing everything that can be theoretically derived, determined by empirical frequencies, or attributed to unrecognizable, ambiguous, and risky situations.

The aim of this article is to attempt to explain, assess and compare the ratio of the probability of erroneous judgments under conditions of uncertainty and comparative analysis, using a procedure for appropriate qualification and quantification of context based on symptoms. The estimated probability amplitudes for each choice vary depending on the specific context, determined by the importance of previous, present, and future symptoms, as well as the sequence of their recognition as subjective images of their objective manifestations. This context assessment procedure is a basic model of the Performance Evaluation Team (PET) method. The PET was developed as an innovative method for human reliability assessment (HRA) [22]. Its algorithm for qualitative and quantitative context assessment is based on symptoms rather than traditional performance shaping factors (PSFs) or performance influencing factors (PIFs). In addition to this conceptually different HRA framework, PET also incorporates models of individual cognition based on the Rasmussen step ladder model (SLM) [23] and the graphical model of group communication [24]. Due to the lack of a generally accepted model of leader decision making, PET assumes that the leader makes a completely reliable judgment if she/he has all the information about the STS. The method has been applied to evaluate and analyze operator performance based on real or simulated scenarios [24] in nuclear and maritime areas. Recent research on the Ellsberg paradoxes, as well as the conjunction and disjunction fallacy, should complement the general conceptual framework of the PET method with a model for analyzing cognition and judgment of a leader or an individual.

The PET procedure uses seven symptom groups for context qualification and quantification: events, goals, parameters, functions, resources, transitions, and actions for describing the context of the entire system [25]. Context is qualitatively and quantitatively determined by the recognized shifted images of non-violated or violated symptoms of the STS and is defined as the object (technology) and subject (human or AI) in situation.

This article proposes two suitable cognitive models that provide a simple explanation for judgmental bias and uncertainty aversion. Symptom recognition is achieved using basic additive and subtractive models that describe the shifts in symptom images. However, in this study of Ellsberg’s color paradoxes [7], the most trivial models are presented, including two- and three-symptom groups without violations for two- and three-color paradoxes, and four-symptom groups with violations for four-color paradoxes, respectively.

The Introduction demonstrates the need to model the construction, implementation mechanism, and control of thought processes, since such a model is absent from the most common version of rational choice/EU theory and its improvements. Furthermore, a scientific and practical approach to eliminating biases and misjudgments related to balancing objective and subjective probabilities is discussed. The purpose of the article is to resolve and explain Ellsberg’s two-, three-, and four-color paradoxes by proposing two models of cognition (additive and subtractive) and using the PET method for quantitative and qualitative assessment of cognitive context based on symptoms.

## 2. An Information Entropy-Based Approach for Holographic STS

### 2.1. PET Hypotheses

Following Pribram’s holonomic model [26] of mental processes (such as perception, memory, cognition, etc.), the PET method applies the analogy between energy and information entropy to the entire STS for the proposed holographic models of thought processes based on the quantification of context as probability [27,28].

To apply the entropy analogy to contextual modeling, several important hypotheses of probabilistic thinking have been formulated:

1. The distinction between macro- and micro-levels and states of the STS. The “holon” of the STS has two levels: “bit” for the macro-level and “quantum” for the micro-level.
2. The STS thinking model consists of numbers of accessible macro/bit and micro/quantum states.
3. The limited number of symptom groups for a specific domain and the number of symptom groups over time.
4. The probabilistic thinking consists of counting and weighing the bits and quantum states of the STS.

This means that subjective probability is assessed at two levels: at the macro-level as the number of contextual alternatives, and each contextual alternative is assessed at the micro-level as the probability amplitude of its occurrence per symptom shift.

### 2.2. PET Context Dynamics Principles

The main principles of the PET dynamic description of the context, as its macro level of approximation, are the following:

∗ Context is a common state of universe (object), mind (subject) in situation;
∗ Context could be described for an isolated system that is unavoidable approximation;
∗ Context consists of associatively relevant elements, which on the macro level should be technologically and organizationally recognized symptoms;
∗ Context can be quantified by accounting the thought or physical accessible states of the STS and subsystems;
∗ Context quantification is not provided for separate subsumed actions but for time interval;
∗ Cognition and context are iterative and recursive;
∗ Cognitive process enables the information to be held in consciousness and discriminates between relevant and irrelevant information, to organize it;
∗ A symptom, after its recognition, could be kept or removed from the human’s context model and memory depending on the situation.

### 2.3. Qualitative Analysis of Context Through Symptoms

Symptoms describe the development of most technological, automatic, manual and mental processes in the STS. They are objective signals/signs/symbols of the STS’s behavior in each situation, insofar as they reflect the properties and state of the object. But since the subject perceives, interprets and recognizes them individually or in groups, they also manifest themselves as subjective images. Symptoms can be placed in the appropriate group according to their distinctive characteristics (for example, the color of a ball).

Usually, symptoms are modeled as indistinguishable (with equal importance) within a group, which facilitates the probabilistic interpretation of the context. But if we have enough information (for example, frequency of occurrence), they can be weighed within the group and between groups.

Each group of symptoms, over time, is described in a column with three sub-columns (*V*, *O* and *S*), where:

∗ *V* represents the number of violations (deviant symptom images) in the group;
∗ *O* is the objective number of symptoms in the group (objectively occurring/measured cues);
∗ *S* is the subjective number of symptoms (subjectively recognized by the operator cue images) in the group.

Each symptom that appears on the object, meaning the addition of 1 to the sub-column with the objective number of symptoms (*O*) of the corresponding group, is recognized by the subject, starting from 0 to the addition of 1 to the sub-column with the subjective number of symptoms (*S*) of this group, in some time interval. The recognition process in this interval is a random variable and can be quantitatively described by a probability distribution function. After the symptom is recognized and accounted for in the context, it can drop out of the short-term memory of the subject (operator) during another time interval, which can also be quantitatively described by a probability distribution function. Usually, two to seven groups of symptoms with their violations are sufficient, with the simplest division into two groups—relevant and irrelevant symptoms.

The objective number of symptoms (*O*), in the PET procedure for qualifying and quantifying the context, at a certain time t for a given group is determined by the registered ones in the real object or in the simulated processes in it. Such examples can be found in [21,24,25]. For Ellsberg’s paradoxes, the moments of time at which the recognition of a symptom (a ball of a certain color) occur are consecutive steps and they are determined by the set conditions of the thought experiment. For the probability distribution functions for recognizing a subjective symptom and disregarding an objective/subjective symptom, the Heaviside step function is used. It is applied to model the transition of recognizing (1) or disregarding (0) (switching between working, short-term and long-term memory) the symptom or the violation of the symptom, which occurs abruptly at a given time *t* due to accumulated knowledge:

$$Symptom(t)=\left\{\begin{array}{cc} 0/1, & t<0\\ 1/0, & t\geq 0 \end{array}\right.$$

or shifted for objective (*O*), subjective (*S*) image or violation of symptom (*V*):

$$\;Symptom(t-t_{O})=\left\{\begin{array}{cc} 0/1, & t<t_{O}\\ 1/0, & t\geq t_{O} \end{array}\right.$$

$$Symptom(t-t_{s})=\left\{\begin{array}{cc} 0/1, & t<t_{s}\\ 1/0, & t\geq t_{s} \end{array}\right.$$

$$Symptom(t-t_{V})=\left\{\begin{array}{cc} 0/1, & t<t_{V}\\ 1/0, & t\geq t_{V} \end{array}\right.$$

The “slack” time of recognition of a given symptom Δ*t* = (*t_O_* − *t_S_*) depends on the type of symptom or violation, as well as on whether the process is of recognition or disregarding. It is usually assumed to range from a few seconds to tens of minutes or even hours. Applications of the PET method often assume that the time can be up to 1 min for Skill-based actions, up to 5 min for Rule-based actions, and up to 30 min for Knowledge-based actions. This assumption is based on the well-known and popular, but refuted, Rasmussen’s SRK model for these three types of actions. However, the minimum duration of such actions has been confirmed by conducting training scenarios on full-scale nuclear power plant simulators [21].

The groups of distinguishable symptoms used to describe the context and to calculate the context probability or contexture, CP, in the nuclear facilities are the following [25]:

1. Parameter (P) is a single parameter or trend of a parameter, and it is a measurable characteristic that allows us to distinguish and determine the state of the STS;
2. Event (E) is a group of properties and a set of results/parameters for the STS occurring in time that have changed its state with probability 1;
3. Function (F) is the purpose of a given equipment of the STS, for which it is designed or exists, for example, a barrier or a safety function; it is also described by a set of parameters (signals) and conditions;
4. Transition (T) is a change/passage from one state (process phase) of the STS to another state (defined in the procedures or models);
5. Goal (G) of a group of tasks is a desired result that the STS (the installation and the operators in each situation) foresees, plans and commits to achieve;
6. Action (A) is an individual or group work/operation that was or must be performed (executed after understanding the context and decision making) by the operator(s), in one or more steps;
7. Resource (R) is a quantitative source of supply (with fluid, energy, people, etc.), support or assistance that can be used when needed;

∗ Violation (V) of symptom is a deviation from the normal way of presenting a symptom by one of the groups (hiding/upsetting/circumventing/distorting the conditions for its control, whereby it becomes difficult to recognize).

### 2.4. Contextual Entropic Model of the PET Method

In the PET method, the contextual entropic model of cognition and judgment means that symptom recognition depends on a cognitive context formed combinatorially and recognized based on previous symptoms step by step, rather than on treating all symptoms as independent. At the small scale, it is a combinatorial model using a probabilistic approach to counting STS states, while at the large scale, it is an entropy model using logarithmic combinatorial calculations of possible STS states.

The key idea is:

(1) $${Context}_{n+1}({Symptom}_{n}|{Context}_{n})\mathrm{or}\;{CP}_{n+1}(S_{n}|{CP}_{n})$$

where:

∗ Context (Symptom) = ordinary Shannon entropy of a symptom source;
∗ ${CP}_{n+1}(S_{n}|{CP}_{n})$ = conditional/contextual entropic given preceding information.

For a random variable Context:

(2) $$Context(S)=-\sum_{s}p(s){log}_{2}p(s)$$

This measures the average uncertainty per symptom. If a source emits Symptoms A and B with probabilities 0.5, then Context (S)=1 bit, because each symptom is equally uncertain.

Real symptoms are usually not independent, e.g., for two-color balls (two symptoms): **red** ball is followed by **red** or **black** ball. And if you know the previous ball was **red**, the uncertainty of the next ball collapses dramatically. So, we use conditional entropy:

(3) $$Context(S_{n}|S_{n-1})$$

This measures uncertainty of the next symptom given the previous one.

For a sequence of symptoms $S_{1},S_{2},S_{3},\ldots \;$the contextual entropy of order k is $Context\;\left(S_{n}|S_{n-1},S_{n-2},\ldots ,S_{n-k}\right)$, which means: *How uncertain is the next symptom if I know the previous* k *symptoms?*

Suppose a sequence of drawn balls alternates perfectly: **black** **red** **black** **red** …

*Naive entropy* ignoring context is used for initial conditions of the cognitive/judgment process: P(red)= P(black)=0.5, so $Context\left(S\right)=1\;\mathrm{bit},$ looks maximally random.

*Contextual entropy* is used for next steps of the cognitive/judgment process:

If you know the previous symptom (ball):

∗ after *O* (objective) symptom always comes *S* (subjective) image because recognition,
∗ after **red** ball, new ones to be checked. Then:

(4) $$Context\;\left(S_{n}|S_{n-1}\right)=Context\;\left(S_{n}|O_{n}\right)=0$$

because the next symptom (ball) is fully determined.

### 2.5. Context Quantification Procedure

Context quantification consists of counting the identical contexts (bit states). The contexture, *CP*(*t*), is obtained as a relation of the number of combinations resulting in the same context to the total number of all context combinations. The PET procedure for evaluation of contexture consists of five steps [21]:

1. Detailed description of the scenario at discrete time intervals (seconds, minutes) in a table with the chronology of its development (normal or emergency) by tracking the change (number) of the emerging, recognized and already reported by the operator symptoms in two to seven groups with three sub-columns for each group;
2. Determination of the number of symptoms of the STS from group *i* (*n*_i_) which arise objectively (*n_oi_*), are recognized subjectively (*n_si_*) or are violated (*n^v^_oi_*) in the scenario;
3. Determination of the initial and boundary conditions. For each normal or emergency scenario *l* (*l* = 1… *L*, *l* is the number in *L* number of scenarios), and for each *k*-operator in the crew (*k* = 1… *K*, *k* is the number of an operator in the crew of *K* operators), it is necessary to specify:

∗ the initial number of subjective symptom images in each group *n_sikl_* = 0, if the operator is not an expert, or
∗ *n^e^_sikl_* if the operator is an expert, *n^e^_sikl_ ≥ n_sikl_* (beginning of group recognition) and
∗ the limiting number of objective symptoms (completion of group recognition) *n_oikl_*, if no violations are available, or
∗ *n^v^_oikl_*, if there are *v* violations for some of the symptoms: *n^v^_oikl_ = v + n_oikl_*.

4. Calculation of cognitive or executive context deviations by the formula:

(5) $$\left|n_{oikl}-n_{sikl}\right|=∆n_{ikl},\;\left|n_{oikl}^{v}-n_{sikl}\right|=∆n_{oikl}^{v},$$

where *i* = 0, 1, 2… *N* are indices of the group of symptoms, *N* is their number, and “*s*” (subjective), “*o*” (objective) and “*v*” (violated) are indices for *S*, *O* and *V*.

5. Calculation of the contexture for cognition and execution, with violation (*CP^v^*) or without violation (*CP*), for each member of the operators’ crew [21]:

(6) $$CP=\frac{Sum\;of\;all\;unknown\;accessible\;states\;of\;the\;STS}{Sum\;of\;all\;possible\;accessible\;states\;of\;the\;STS}$$

(7) $${CP}^{v}\left(t\right)=1-\frac{\prod_{i=1}^{N}\left[n_{i}^{s}\left(t\right)+1\right]}{\prod_{i=1}^{N}\left[n_{i}^{vo}\left(t\right)+1\right]}$$

(8) $$CP\left(t\right)=1-\frac{\prod_{i=1}^{N}\left[n_{i}^{s}\left(t\right)+1\right]}{\prod_{i=1}^{N}\left[n_{i}^{o}\left(t\right)+1\right]}$$

Section 2 formulates the hypotheses, principles, contextual entropic model and symptom-based context assessment procedure within the information entropy-based PET approach for the holographic description of a socio-technical system.

## 3. Creating Step-by-Step Models of Biased Cognition

### 3.1. Counting Symptoms to Synthesize Thought Processes

Explaining more complex concepts and ideas in simpler, more understandable terms involves generalizing and filtering information, representing a cognitive process that reduces the entropy of that information. For example, the distinction between “bars and dots” or “counting beans, putting them in, and taking them out” from different pots, as the Mayan priests did [3], can also be applied to symptom groups to identify, distinguish, and calculate alternative states of the STS [29].

According to thermodynamic principles, decreasing the information entropy of an STS requires an open system, allowing information to be transferred, or work to be performed to “cool” the STS: transfer knowledge from working memory to short-term and long-term memory, increase structural order, or remove symptoms. As local entropy decreases, the entropy of the surrounding environment must increase by a greater amount, thereby increasing the overall entropy of the universe. This can be achieved by adding more information to working memory, as well as by removing unnecessary, irrelevant, or already moved recognized information from working to short-term or long-term memory.

Based on this idea, we can construct a very simple model for a non-additive approach to cognition, which is a well-established fact in cognitive theory but has not been tested or clearly demonstrated using simple subtraction in previous experimental studies.

The idea behind the PET method is that the hypothetical cognitive process occurs in several discrete steps of approximation of the subjectively recognized number of symptoms (S_i_) to the objective number of symptoms (O_i_) of the group i = 1… I.

The objective number of symptoms may change in a stepwise manner over time. The steps in each column and sub-column can be performed sequentially, simultaneously, or with a delay. A column with violations for each group can also be added. The PET method uses seven-symptom groups, I = 7. However, the examples here are of the simplest models of cognitive biases and ambiguity aversion, so we have only two-, three- and four-symptom groups, I = 2, I = 3 or I = 4, respectively.

### 3.2. Step-by-Step Models of Additive–Subtractive Cognition

To explain and address various cognitive biases using the PET method, two basic models were applied to define and assess their cognitive context:

1. Stepwise Additive Cognition with memorizing (SAC_m_) in two steps—without forgetting recognized symptoms O_i_ and S_i_ (while preserving their number). The SAC_m_ model is presented in Table 1.
2. Stepwise Subtractive Cognition with disregarding (SSC_d_) in four steps—with disregard of the already recognized O_i_ and S_i_ symptoms (numbers of both decrease, but with a shift). The SSC_d_ model is presented in Table 2.

Description and meaning of cells in Table 1 and Table 2: Table 1 and Table 2 show three groups of symptoms (balls of a certain color—black, red and green), which are used below to model and solve the two-color and three-color Ellsberg paradoxes. When modeling these two paradoxes, there is no violation and therefore sub-column V is omitted.

The steps of group A (1A, 2A, 3A and 4A), or those for groups B and C, show the number of steps of symptom recognition for the respective model (two for the additive and four for the subtractive model). The number of “objective” symptoms/balls, O, of the respective color is indicated by n, N − n or N/2, depending on how many of them are in the respective paradox (white for two-color and grey for three-color Ellsberg paradox). And during recognition, the number of recognized “subjective” symptoms, S, increases by one (for clarity, the cell is colored in the corresponding color), since the balls are drawn sequentially one by one and would be of the corresponding color. The sequence black, red, and green is conditional, i.e., their places can be swapped.

Section 3 presents the two-step additive and four-step subtractive models of cognition, which are used in the subsequent Section 4 to resolve and explain Ellsberg’s two-, three-, and four-color paradoxes.

## 4. Resolved and Explained Cognitive Biases

The PET procedure for all biased judgments under study is performed in three stages:

1. A description of the biased decision-making problem is provided.
2. The contextual models used to determine the compared subjective probabilities of a successful judgment, and the ratio between them, and to resolve bias are presented in tables that reflect the situation if they differ from the SAC_m_ and SSC_d_ models.
3. The obtained PET results—point estimates—are compared and analyzed.

### 4.1. Ellsberg’s Two-Color Problem

Ellsberg’s two-color paradox involves two urns each containing N = 100 balls of two colors (red and black). The ‘clear’ urn contains N/2 black and N/2 red balls, whereas the ‘vague’ urn contains n black and (N-n) red balls in an unknown proportion, where n ϵ [0, N]. If the color that the player draws is the same as she/he predicted, then the player will win $Z (Z > 0), otherwise nothing.

Suppose players are offered three games to be played as follows:

1. Game 1 (noncomparative): Players must guess the color (red or black) and then choose a ball from the ‘clear’ urn.
2. Game 2 (noncomparative): Player must guess the color (red or black) and then choose a ball from the ‘vague’ urn.
3. Game 3 (comparative): Player must guess the color (red or black) and then choose a ball from the preferred ‘clear’ vs. ‘vague’ urn.

The color, numbers of balls, bets and games are shown in Table 3.

*Example. How to apply the context quantification procedure to Ellsberg’s two-color paradox*

This procedure consists of counting the identical contexts. The contexture, *CP*(*t*), is obtained as the ratio of the number of combinations leading to the same context to the total number of all possible context combinations. The PET procedure consists of five steps, which are greatly simplified for the Ellsberg two-color paradox:

1. Ellsberg’s two-color problem involves two urns each containing balls of two colors (e.g., red and green). Both urns contain two balls.
2. Let us consider that the two urns (*i* = 2) contain two balls each, *n_o_*_1_ *= n_o_*_2_ = 2, but the subjectively recognized balls under the conditions of Ellsberg’s thought experiment are *n_s_*_1_ *= n_s_*_2_ = 1.
3. The initial and boundary conditions of recognition are 0 and 1.
4. The deviations calculated by Formula (5) for *i* = 1 or 2 are:

(9) $$\left|n_{oi}-n_{si}\right|=1$$

**Figure 1:**
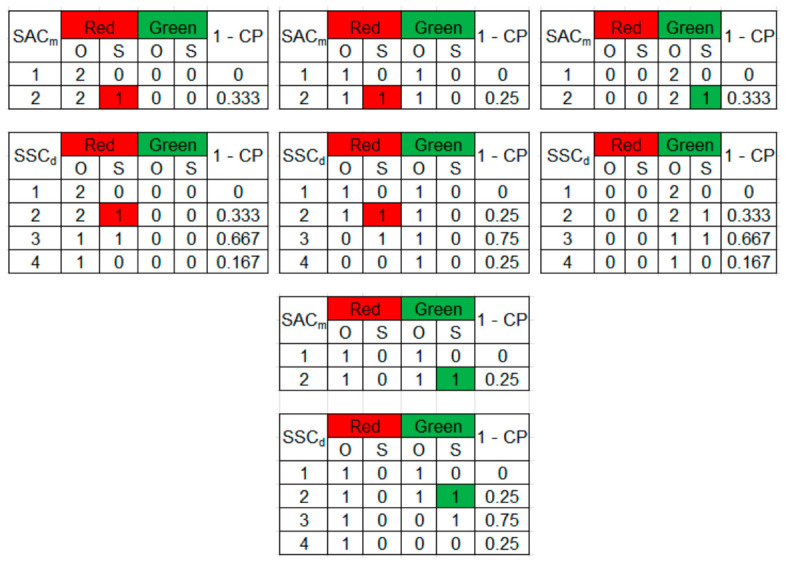
Models and results of an application of the PET procedure to Ellsberg’s two-color paradox.

5. The contexture calculation (*CP*) is done for each row of the following four possible cognitive processes with both models SAC_m_ and SSC_d_, shown in Figure 1 below.

Applying Formula (6) we will get the following results, which we need to sum up for the corresponding step and model, shown in Figure 1 below.

The principal and the PET results for the ratios of the OP and of the SP of a successful judgment for choosing a ball from the ‘clear’ urn and the ‘vague’ urn for the noncomparative cases (games 1, 2) and for the comparative case (game 3) are presented in Table 4. Most people seem indifferent to betting on **red** or **black** for either urn in the noncomparative games, yet they prefer to bet on the ‘clear’ urn rather than on the ‘vague’ urn with the unknown composition in the game 3 comparative case [30].

To resolve Ellsberg’s two-color paradox, we apply the models for two-symptom groups from Table 1 and Table 2. Using the PET procedure for calculating context probability (CP) or contexture, we can calculate the probabilities of successful judgment and ratio between them, the lower bound of which is SP = (1 − contexture) at all steps of the cognitive process. The PET method allows us to obtain more optimistic predictive results using various models of the reliability of individual cognition based on Rasmussen’s stepladder model [24]. In this study, the results of the Task Definition and Activation individual reliability model were used, which corresponds to the median SP. For games 1 and 2, the SAC_m_ model is used, and for game 3, the SSC_d_ model. Using these models, we obtain the following results for the SP of successful judgment, presented in Table 4.

The preference results and SP values obtained using the PET method confirm Ellsberg’s conclusion that the SP of drawing a red or black ball from the “clear” urn are higher (0.28) than in the “vague” urn (0.21). However, they also show that the sum of the SPs in both urns is less than 1, even though these are complementary events. These results contradict Savage’s ST principle [5], which is equivalent to the independence axiom of the EU hypothesis.

### 4.2. Ellsberg’s Three-Color Problem

The well-known Ellsberg’s three-color paradox is a sufficiently representative case for the solutions proposed by the PET method [31].

In its classic version, there is an urn containing three colors of balls. The urn consists of two parts containing (N + N/2) = 90 balls: a ‘clear’ part with N/2 = 30 **green** (in Ellsberg’s description it is red) balls, and the remaining ‘vague’ part with N = 60 balls contains (N-n) **red** and n **black** (in Ellsberg’s description it is yellow) colors in an unknown proportion, where n ≤ [0, N]. A single ball is drawn from the urn.

The player is offered to bet on one of the following: bet1, bet2, bet3, or bet4, defined in Table 5. If the player’s drawn color matches the predicted color, she/he wins $Z (Z > 0); otherwise, she/he wins nothing.

Suppose players are offered two games as follows:

1. Comparative objective–subjective game. The player must guess the color and bet on **green** (b1) vs. **red** (b2), then select a ball from the urn.
2. Comparative subjective–objective game. The player must guess one of two colors, **green**/**black** (b3) vs. **red**/**black** (b4), then select a ball from the urn.

If the color that the player draws is the same as she/he predicted, then the player will win a = $2 × Z (Z > 0); otherwise, she/he will win nothing, b = $0 (in Ellsberg’s experiment a = $100, b = $0). To simplify the task of the four-color problem of Ellsberg and Machina, the unit of payoff Z is chosen to be the least common multiple of Machina’s experiments (Z = $4000). The unit of payoff Z is equivalent to one symptom (ball). In the four-color problem of Ellsberg, Z = $50 (80 times less), otherwise, she/he wins nothing.

Table 6 compares the probabilities of a player’s judgments, for which OP is determined by the minimum chance of each event occurring, and SP is determined by the principle of indifference (insufficient reason) [31].

In the experiments conducted by Ellsberg and after him, people prefer to bet on the **green** (bet 1) over the **red** (bet 2) ball, and to bet on “**red** or **black**” (bet 4) in front of “**green** or **black**” (bet 3) balls. This preference cannot be explained by the EU hypothesis. The first choice complies with the ST principle, but the second choice violates it, which requires that the order of bets 1 through 2 be preserved in bets 3 and 4 (since the two pairs differ only in the payoff for drawing a **black** ball, which is constant for each pair). Nevertheless, these choices are intuitive: bet 1 offers the Z prize with an objective likelihood OP_bet1_ = 1/3 and bet 2 offers the same prize but in an element of the subjective partition and ambiguity SP_bet2_ < 1/3 (**red**, **black**), if the game is fair. In the same way, bet 4 offers the prize with an objective likelihood OP_bet4_ = 2/3, whereas bet 3 offers the same payoff with subjective likelihood SP_bet3_ < 2/3 on union of the unambiguous event **green** and the ambiguous event **black**.

As with Ellsberg’s two-color paradox, the preference model contradicts the EU hypothesis because it implies that the vague SP_bet2_ of **red** is less than the OP_bet1_ of **green** (SP_bet2_ < OP_bet1_ = 1/3) and the vague SP_bet3_ with unknown proportion, between ‘**green** or **black**’ balls, is less than the OP_bet4_ of ‘**black** or **red**’ (SP_bet3_ < O_bet4_ = 2/3). Therefore, the sum of SP_bet2_ + SP_bet3_ < 1 for these outcomes in the decision-making process is not equal to one, although these are complementary events.

Ellsberg found that decision makers prefer bets with higher OPs, other things being equal, in those situations where the combined probabilities and payoffs are the same in the urns. He found that people are prone to ambiguity.

To solve Ellsberg’s three-color paradox, we apply the two cognitive context models, simulated with the PET context quantification procedure, based on Table 1 for additive cognition with memorizing and Table 2 for subtractive cognition with disregarding. For non-comparative game 1 the SAC_m_ model is used, and for comparative game 2 the SSC_d_ model is used. With their help, we can obtain the following results, shown in Table 7. In this study, as well as for Ellsberg’s two-color paradox, the results for the Task Definition and Activation individual reliability model were used, which corresponded to the median SP.

Comparisons between the principle and the PET results of the Ellsberg three-color paradox are added and presented in Table 6 too.

### 4.3. Ellsberg’s Four-Color Problem

#### 4.3.1. Four-Color Ellsberg Problem Description

The example, known as the four-color Ellsberg paradox, is presented in [7] (p. 654, note 4). It involves an urn containing balls of four colors (e.g., black, red, green, and yellow). The urn contains 2 × N balls (200 balls in Ellsberg’s experiment), where n black, N − n red, N/2 green and N/2 yellow balls, where n ϵ [0, N]. It means that the vague part of the urn contains n black and (N − n) red balls in an unknown proportion. One ball will be drawn from the urn. A person is asked to bet on one of the acts b1, b2, b3 and b4 defined in Table 8.

Suppose players are offered two games to be played as follows:

1. Comparative objective–subjective game. The player must guess the color and bet on black/red (b1) vs. black/green (b2), then select a ball from the urn.
2. Comparative subjective–objective game. The player must guess one of two colors, red/yellow (b3) vs. green/yellow (b4), then select a ball from the urn.

The choice of Z is subjective for each agent. Varying Z can also change the direction of the ambiguity aversion, as in the examples of Ellsberg (by modulating the waves of symptoms). If ambiguity is important to the decision maker, the magnitude of the difference between the subjective Z of two agents can determine whether it will compensate for the differences in the subjectively estimated probability measures from the different numbers of balls in the experiments. This allows us to use a dimensionless quantity by adding an additional symptom-wave; it is a third column (V_i_—violation column, i = 1… 4) of the number of balls of a certain color.

The original PET method is based on modeling symptom distortion (violation), which reduces contexture and the human error probability (HEP). But the peculiarity of modeling the payoff matrix is that this wave should have the opposite effect, increasing the probability of making a given judgment. Therefore, the violation should be placed on the color of ball that pays less after drawing, based on the symmetry of the payoff matrix (Table 8, where Z_ij_, j = 1… 4).

#### 4.3.2. Principal Results

Ellsberg’s four-color problem, as well as Ellsberg’s two-color [29] and three-color [31] problems, show that individuals’ preferences deviate from the classical SEU model by exhibiting a systematic preference for objective over subjective bets, and typical rankings bet 1 > bet 2 (OP_b1_ > SP_b2_) and bet 3 < bet 4 (SP_b3_ < OP_b4_) respectively reveal the same type of a phenomenon known as ambiguity aversion. Ellsberg observed that such examples can be viewed as providing STS violations of Savage’s ST principle, postulate P2 [5] (p. 23). Such examples have stimulated the development of alternatives to the SEU model, most notably the rank-dependent expected utility or Choquet expected utility model, which describes preferences for subjective bets.

#### 4.3.3. Performance Evaluation of Teamwork Results

**Figure 2:**
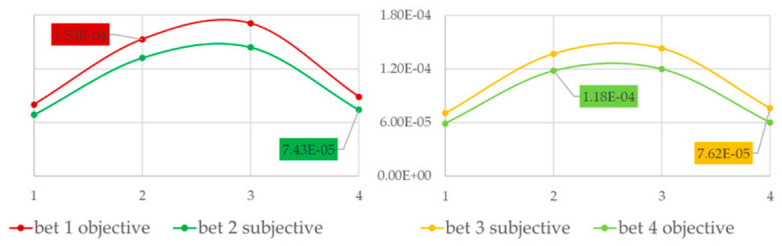
The PET stepwise cognition for b1 vs. b2 and b3 vs. b4 for the (100 + 100),2 case of Ellsberg’s original four-color paradox.

The results of PET and the comparison of bets for the ratio of the sums of subjectively estimated probability measures of a successful decision to select a ball from the “clear” and “vague” parts of the urn for the comparative objective–subjective (b1 vs. b2) and subjective–objective (b3 vs. b4) games are presented in Table 9 and Table 10 and Figure 2 for the original four-color Ellsberg’s paradox (100 + 100),2 [32].

As the number of balls increases, the inequalities remain valid only for the SSC_d_ model, which, due to the presence of subjective recognition, ends at the fourth step of cognition. Of course, there is always the possibility that it ends at a previous step or a next step and that the four-color Ellsberg paradox is not observed. The value of Z is highly individual and here the dependence on its absolute value is not studied, but only the ratio between the payoffs for the different ball colors.

By using the PET procedure to calculate CP or contexture, we can calculate the sums of subjectively estimated probabilities as a ratio for a successful judgment. These are determined by a conditionally alternative distribution of balls, actions, and bets, the lower bound of which is SP = (1 − CP).

The choice preference results and SP values obtained using the PET method confirm Ellsberg’s conclusion of ‘clear’ over ‘vague’ bets and typical rankings bet 1 > bet 2 and bet 3 < bet 4, as follows from Table 10.

*Description and meaning of the cells in Table 9 and Table 10:* Table 9 presents the sums of the results for all (101 in number) probability amplitudes (per ball) of the variants of drawing the ball from the respective color. And in Table 10 these results are summed up to show the probability for the respective bets on two of the colors (b1, b2, b3 or b4). 

In Section 4, Ellsberg’s color paradoxes with two, three, and four colors are solved and explained. The principal results for the ratio between objective and subjective probability in them are shown, as well as the quantitative results with the PET method, which confirm and explain them based on the proposed additive and subtractive models of cognition.

## 5. Discussion

OPs and SPs used to explain uncertainty (risk and ambiguity) of decisions in [2,5], as well as the numerous complementary and corrective models after them, are related to utility and do not describe the construction of cognitive processes. Modeling decision-making processes should be associated with changes in the information entropy [27].

A decision maker must clearly distinguish not only between “task-irrelevant” and “task-relevant” stimuli, compare them together in “clear and vague perspectives” [30], and possibly recognize them sequentially, simultaneously, or in a mixed manner, but also ignore them after recognition in the process of cognition from use.

Therefore, to avoid distortion of these cognitive processes, they must be identified, interpreted, qualified, and quantified in the context of the entire STS.

This conditional sequence of symptom recognition is used in the proposed model of cognition and judgment using the PET method:

(1) Distinguishing relevant and irrelevant symptoms (two-valued logic for Ellsberg paradoxes);

(2) Comparing and applying sequential, parallel and/or mixed cognition to the clear and ambiguous perspectives for a probabilistic evaluation;

(3) In addition, for already recognized symptoms, the person shortens the options by removing already recognized symptoms, as is done in 2 to 4.

This way of arranging the steps and recognizing the context is not universal and for other cases three-valued logic or more steps are required, using the third sub-column in the PET method (violations), which also distorts the thought processes.

STS states depend primarily on “task-relevant” symptoms, but they are perceived by the relevant intellect as subjective images when compared with existing objective ones with a certain uncertainty.

Developing a unified framework for explaining uncertainty is an open problem in decision making. It is crucial for avoiding uncertainty in judgment, risk management, and communication. The imaginary framework of the two- and four-step cognitive process proposed here resembles two-stroke and four-stroke internal combustion engines, but the development, details, and optimization of the thought processes are not entirely clear. 

Explaining and resolving uncertainty is also a way to overcome the widely used expert judgment of HEP in HRA. The use of PET models for stepwise symptom recognition allows for a unified and reasoned explanation and resolution of biased judgments in uncertain and comparative contexts. The PET method was successfully applied not only to Ellsberg’s two-, three- and four-color paradoxes discussed in this article, but also to the explanation and resolution of the Machina paradoxes and the logical fallacies of conjunction and disjunction. This opens new opportunities to consistently and systematically address a multitude of different biases that hinder rational decision making by humans.

Section 5 discusses ways of explaining and resolving decision-making problems under comparison and uncertainty and proposes to use the step-by-step approach of the PET method to provide a unified and valid explanation and resolution of biased judgments, without necessarily making it universal and applicable to all such paradoxes.

## 6. Conclusions

1. When choosing between “clear” and “vague” sets, the “clear” choice is made in two steps, and the “vague” in four steps:
  - The two-step additive model SAC_m_ is used for cognitive processes without comparison.
  - The four-step subtractive model SSC_d_ is used for cognitive processes involving comparison.
2. The SP of a successful judgment is smaller than the OP at all steps of the cognitive process if there are no symptoms with violations.
3. Typically, the sum of the SPs of complementary events is less than 1.
4. Judgment in an unambiguous context is a monotonic process of increasing contexture, and judgment in an ambiguous comparative context is a non-monotonic, wave-like process caused by the interfering sum of the amplitudes of the probability of recognized symptoms [14].

These examples based on the Ellsberg paradoxes demonstrate that contextual entropic models of additive and subtractive cognition can explain the uncertainty in comparison settings and the paradoxes found in the expected utility model and its derivatives.

The problem with paradoxes is that they represent a mixture of “measurable” (risk) and “immeasurable” (ambiguity) uncertainty. Due to the peculiarities of human cognition, the utility reference point, where we have no losses or gains (see “Prospect Theory Graph” [33]), generates ambiguity shifts such that the probability of a choice’s preference (accounted for by utility) significantly influences the subjective success probability (corresponding to objective probability). This can be explained not only by the utility function proposed by Bernoulli and presented in various modern forms, but also by considering expected utility as a symptom and incorporating its non-monotonic effects along with other symptoms in the contextual entropic model.

Section 6 presents conclusions regarding the results and prospects for applying the PET method to address and explain paradoxes associated with uncertainty in decision making. It is concluded that by treating expected utility as a symptom and incorporating its non-monotonic effects, along with other symptoms, into a contextual entropy model, the PET method offers a viable alternative to the utility function widely used from Bernoulli to the present day.

## Figures and Tables

**Table 1:** Stepwise Additive Cognition with memorizing (SAC_m_) *.

Groups	A		B		C	
Steps	**O**	**S**	**O**	**S**	**O**	**S**
1A	n	0	N − n	0	N/2	0
2A	n	1	N − n	0	N/2	0
1B	n	0	N − n	0	N/2	0
2B	n	0	N − n	1	N/2	0
1C	n	0	N − n	0	N/2	0
2C	n	0	N − n	0	N/2	1

* The background colors of the headings and individual cells of Table 1 and Table 2 have the following meaning: black, red, and green are the colors of the balls for the two- and three-color Ellsberg paradoxes. The common white background color (left) refers to the two- and three-color Ellsberg paradox, while the added gray background color (right) refers only to the three-color Ellsberg paradox. The white color of the letters and numbers used in Table 1 and Table 2 is necessary so that they can be better distinguished from the background color. Same as Table 2.

**Table 2:** Stepwise Subtractive Cognition with disregarding (SSC_d_) *.

Groups	A		B		C	
Steps	**O**	**S**	**O**	**S**	**O**	**S**
1A	n	0	n	0	N/2	0
2A	n	1	N − n	0	N/2	0
3A	n − 1	1	N − n	0	N/2	0
4A	n − 1	0	N − n	0	N/2	0
1B	n	0	N − n	0	N/2	0
2B	n	0	N − n	1	N/2	0
3B	n	0	N − n − 1	1	N/2	0
4B	n	0	N − n − 1	0	N/2	0
1C	n	0	N − n	0	N/2	0
2C	n	0	N − n	0	N/2	1
3C	n	0	N − n	0	N/2 − 1	1
4C	n	0	N − n	0	N/2 − 1	0

**Table 3:** Number, color and payoff matrix of balls for the Ellsberg two-color problem.

Bet and Game	‘Clear’ Urn		‘Vague’ Urn	
Color	Black	Red	Black	Red
Number of Balls	N/2	N/2	n	N − n
bet 1, game 1	Z	0	-	-
bet 2, game 1	0	Z	-	-
bet 3, game 2	-	-	Z	0
bet 4, game 2	-	-	0	Z
bet 5 is bet 1 or bet 2, game 3	Z	0	Z	0
bet 6 is bet 3 or bet 4, game 3	0	Z	0	Z

**Table 4:** Principle and PET results of Ellsberg’s two-color problem.

Game	‘Clear’ Urn			Ratio	‘Vague’ Urn				
results	PET SAC_m_ steps		principal			PET SSC_d_ steps			
1 black	0.5		OP_c1_	=	OP_v1_	0.5			
1 red	0.5		OP_c1_	=	OP_v1_	0.5			
2 black	0.5	0.28	SP_c2_	≈	SP_v2_	0.5	0.28	0.33	0.21
2 red	0.5	0.28	SP_c2_	≈	SP_v2_	0.5	0.28	0.33	0.21
3 black	0.5		OP_c3_	=	OP_v3_	0.5			
3 red	0.5		OP_c3_	=	OP_v3_	0.5			
3 black	0.5	0.28	SP_c3_	>	SP_v3_	0.5	0.28	0.33	0.21
3 red	0.5	0.28	SP_c3_	>	SP_v3_	0.5	0.28	0.33	0.21

**Table 5:** Number of balls, color, and payoff matrix for the three-color problem.

Bet and Game	‘Clear’ Part	‘Vague’ Part	
Color	green	black	red
Number of Balls	N/2	n	N − n
bet 1, game 1	Z	0	0
bet 2, game 1	0	0	Z
bet 3, game 2	Z	0	Z
bet 4, game 2	0	Z	Z

**Table 6:** Principle and PET results of Ellsberg’s three-color problem.

Games	Unambiguous Event	Ratio	Ambiguous Event
Principle comparisons			
game 1: bet 1 vs. bet 2	OP_bet1_ = 0.333	>	SP_bet2_
game 2: bet 3 vs. bet 4	OP_bet4_ = 0.333	>	SP_bet2_
PET comparisons			
game 1: bet 1 vs. bet 2	OP_bet1_ = 0.333	>	SP_bet2_ ≈ 0.293
game 2: bet 3 vs. bet 4	OP_bet4_ = 2/3	>	SP^max^_bet3_ ≈ 0.605 = 0.303 + 0.302

**Table 7:** PET results of the Ellsberg three-color problem.

Results	PET SAC_mi_, i = 1, 2 Steps		PET *SSC_dj_*, j = 1, 2, 3, 4 Steps			
Green	0.147	0.293	0.147	0.293	0.303	0.151
Red	0.147	0.293	0.147	0.293	0.302	0.151
Black	0.147	0.293	0.147	0.293	0.302	0.151
SUM	0.441	0.879	0.441	0.879	0.907	0.453

**Table 8:** Number of balls, color, and payoff matrix for the four-color problem.

Bet and Game	‘Vague’ Part		‘Clear’ Part	
Color	Black	Red	Green	Yellow
Number of Balls	n	N − n	N/2	N/2
bet 1, game 1	2 × Z	2 × Z	0	0
bet 2, game 1	2 × Z	0	2 × Z	0
bet 3, game 2	0	2 × Z	0	2 × Z
bet 4, game 2	0	0	2 × Z	2 × Z

**Table 9:** The PET results for the (100 + 100),2 Ellsberg’s four-color paradox *.

Model	b1	b2	b3	b4
Step	SSC_d_	SSC_d_	SSC_d_	SSC_d_
Step 1 SAC_a_ = SSC_d_	3.990067 × 10^−5^	3.423627 × 10^−5^	3.524137 × 10^−5^	2.942610 × 10^−5^
	3.990067 × 10^−5^	3.423627 × 10^−5^	3.524137 × 10^−5^	2.940633 × 10^−5^
	3.990067 × 10^−5^	3.423627 × 10^−5^	3.5234137 × 10^−5^	2.942610 × 10^−5^
	3.990067 × 10^−5^	3.423627 × 10^−5^	3.524137 × 10^−5^	2.942610 × 10^−5^
Step 2 SAC_a_ = SSC_d_	7.627661 × 10^−5^	6.488070 × 10^−5^	6.926176 × 10^−5^	5.760796 × 10^−5^
	7.627661 × 10^−5^	6.725155 × 10^−5^	6.689081 × 10^−5^	5.756842 × 10^−5^
	7.980135 × 10^−5^	6.847254 × 10^−5^	7.048275 × 10^−5^	5.8852195 × 10 ^−5^
	7.980135 × 10^−5^	6.847254 × 10^−5^	7.048275 × 10^−5^	5.885219 × 10^−5^
Step 3 SSC_d_	8.413639 × 10^−5^	7.270089 × 10^−5^	7.239611 × 10^−5^	6.062011 × 10^−5^
	8.641782 × 10^−5^	7.115504 × 10^−5^	7.700637 × 10^−5^	6.138978 × 10^−5^
	8.133599 × 10^−5^	6.894199 × 10^−5^	7.183718 × 10^−5^	6.002924 × 10^−5^
	8.133599 × 10^−5^	6.978932 × 10^−5^	7.189206 × 10^−5^	6.002924 × 10^−5^
Step 4 SSC_d_	4.383057 × 10^−5^	3.814636 × 10^−5^	3.680855 × 10^−5^	3.093217 × 10^−5^
	4.497128 × 10^−5^	3.618801 × 10^−5^	4.029910 × 10^−5^	3.131700 × 10^−5^
	4.066799 × 10^−5^	3.492099 × 10^−5^	3.591909 × 10^−5^	3.001462 × 10^−5^
	4.066799 × 10^−5^	3.489466 × 10^−5^	3.594603 × 10^−5^	3.001462 × 10^−5^

* The background colors in Table 9 are the results for each ball: black, red, green, and yellow.

**Table 10:** The PET comparison for the (100 + 100),2 case of Ellsberg’s four-color paradox *.

Bet/Steps	Step 1	Step 2	Step 3	Step 4
bet 1 objective	7.98 × 10^−5^	1.53 × 10^−4^	1.71 × 10^−4^	8.88 × 10^−5^
bet 2 subjective	6.85 × 10^−5^	1.32 × 10^−4^	1.44 × 10^−4^	7.43 × 10^−5^
bet 3 subjective	7.05 × 10^−5^	1.37 × 10^−4^	1.43 × 10^−4^	7.62 × 10^−5^
bet 4 objective	5.89 × 10^−5^	1.18 × 10^−4^	1.20 × 10^−4^	6.00 × 10^−5^
Model	SAC_a_ = SSC_d_	SAC_a_ = SSC_d_	SSC_d_	SSC_d_

* In Table 10 an attempt is made to illustrate the color pairs respectively: for b1—**black**/**red** with dark **red**; for b2—**black**/**green** with dark **green**; for b3—**red**/**yellow** with **orange**; and for b4—**green**/**yellow** with light **green**. Due to the use of different color gamuts in different processing tools, these colors may not correspond to the authors’ intentions.

## Data Availability

Dataset available on request from the author.

## Abbreviations

The following abbreviations are used in this manuscript:

CP	Context Probability (contexture)
EU	Expected Utility
HEP	Human Error Probability
HRA	Human Reliability Assessment
O_i_	Objective number of symptoms of group i
OP	Objective Probability
PET	Performance Evaluation of Teamwork
SAC_m_	Stepwise Additive Cognition with memorizing
SEU	Subjective EU
S_i_	Subjective number of symptoms of group i
SP	Subjective Probability
SSC_d_	Stepwise Subtractive Cognition with disregarding
ST	Sure-Thing
STS	Socio-Technical System
V_i_	number of Violated symptoms of group i
Z	Payoff
